# Real-life clinical management patterns in extensive-stage small cell lung cancer across France: a multi-method study

**DOI:** 10.1186/s12885-024-12117-9

**Published:** 2024-04-05

**Authors:** Bertrand Mennecier, Jonathan Khalifa, Renaud Descourt, Laurent Greillier, Charles Naltet, Lionel Falchero

**Affiliations:** 1 Department of Thoracic Oncology, Strasbourg University Hospital, Strasbourg, France; 2 Department of Radiation Oncology, Claudius Regaud Institute, Cancer University Institute of Toulouse Oncopole, Toulouse, France; 3 Department of Medical Oncology, Augustin-Morvan Hospital, Brest University Hospital, Brest, France; 4 Aix Marseille University, APHM, INSERM, CNRS, CRCM, Hôpital Nord, Multidisciplinary Oncology and Therapeutic Innovations, Marseille, France; 5 Department of Thoracic Oncology & CIC, Paris Saint Joseph Hospital, 1425/CLIP2 Paris-Nord, Paris, France; 6 Department of Pulmonology and Thoracic Oncology, North West Hospital of Villefranche, Villefranche, France

**Keywords:** Small cell lung cancer, Extensive-stage, Clinical management, Immunotherapy, First-line therapy

## Abstract

**Background:**

We designed this study based on both a physician practice survey and real-world patient data to: (1) evaluate clinical management practices in extensive-stage small cell lung cancer (ES-SCLC) among medical centers located across France; and (2) describe first-line treatment patterns among patients with ES-SCLC following the introduction of immunotherapy into clinical practice.

**Methods:**

A 50-item questionnaire was completed by physicians from 45 medical centers specialized in SCLC management. Responses were collected from June 2022 to January 2023. The survey questions addressed diagnostic workup of ES-SCLC, chemoimmunotherapy in first-line and second-line settings, and use of prophylactic cranial irradiation (PCI) and radiotherapy. In parallel, using a chart review approach, we retrospectively analyzed aggregated information from 548 adults with confirmed ES-SCLC receiving first-line treatment in the same centers.

**Results:**

In ES-SCLC, treatment planning is based on chest computed tomography (CT) (as declared by 100% of surveyed centers). Mean time between diagnosis and treatment initiation was 2–7 days, as declared by 82% of centers. For detection of brain metastases, the most common imaging test was brain CT (84%). The main exclusion criteria for first-line immunotherapy in the centers were autoimmune disease (87%), corticosteroid therapy (69%), interstitial lung disease (69%), and performance status ≥ 2 (69%). Overall, 53% and 36% of centers considered that patients are chemotherapy-sensitive if they relapse within ≥ 3 months or ≥ 6 months after first-line chemoimmunotherapy, respectively. Among the 548 analyzed patients, 409 (75%) received chemoimmunotherapy as a first-line treatment, 374 (91%) of whom received carboplatin plus etoposide and 35 (9%) cisplatin plus etoposide. Overall, 340/548 patients (62%) received maintenance immunotherapy. Most patients (68%) did not receive radiotherapy or PCI.

**Conclusions:**

There is an overall alignment of practices reflecting recent clinical guidelines among medical centers managing ES-SCLC across France, and a high prescription rate of immunotherapy in the first-line setting.

**Supplementary Information:**

The online version contains supplementary material available at 10.1186/s12885-024-12117-9.

## Introduction

Small cell lung cancer (SCLC) is a poorly differentiated high-grade neuroendocrine carcinoma that accounts for approximately 15% of all lung cancer cases and that is highly related to tobacco smoking [1–3]. The clinical management of SCLC is difficult due to the aggressive nature of the disease, as SCLC is characterized by a rapid doubling time, high growth fraction, and early development of widespread metastases [2]. There are also several poor prognostic factors in SCLC, which include poor performance status (PS), weight loss, increased age, male sex, elevated lactate dehydrogenase, and hyponatremia [1]. SCLC is typically classified into two stages: limited-stage and extensive-stage (ES) disease. According to the tumor, node, metastasis (TNM) staging system from the American Joint Committee on Cancer (AJCC), ES-SCLC is defined as stage IV disease (any T, any N, M1a/b) or T3–4 due to involvement of multiple lung nodules [4]. At diagnosis, most SCLC cases (70%) present as ES disease [5]. In France, there are limited data regarding the epidemiology of ES-SCLC. However, the estimated incidence rates of SCLC in 2018 in France were 5.5 and 2.7 per 100,000 person-years, in men and women, respectively [6]. The 5-year net survival for SCLC was also estimated at 7% among those diagnosed between 2010 and 2015 [7].

Until recently, the frontline standard of care for ES-SCLC was platinum-based doublet chemotherapy with or without prophylactic cranial irradiation (PCI) and with or without consolidative thoracic radiotherapy. Consolidative thoracic radiotherapy could be administered in selected patients with residual thoracic disease and low-bulk extrathoracic metastases who show a tumor response after initial systemic treatment. Platinum-based chemotherapy involves 4 to 6 cycles of cisplatin or carboplatin plus etoposide [3]. Despite the high chemosensitivity of SCLC, most patients with ES-SCLC relapse within 6 months [1].

To enhance frontline treatment with chemotherapy, the programmed death-ligand 1 (PD-L1) inhibitors, atezolizumab and durvalumab, received regulatory approval in 2019 and 2020, respectively, in combination with chemotherapy, for the first-line treatment of adult patients with ES-SCLC, based on the respective phase III trials: IMpower133 [8] and CASPIAN [9]. Updated overall survival (OS) data from these two trials demonstrated a 2-months OS improvement with chemoimmunotherapy compared to chemotherapy alone [10, 11]. Hence, clinical practice guidelines currently recommend, for eligible treatment-naïve patients with ES-SCLC, with a PS of 0–1, and no contraindications to immunotherapy, two first-line therapy regimens: atezolizumab plus carboplatin plus etoposide (4 cycles) as induction therapy followed by maintenance atezolizumab, and durvalumab plus etoposide plus carboplatin/cisplatin (4 cycles) as induction therapy followed by maintenance durvalumab [1, 2, 12]. Maintenance immunotherapy is restricted to patients who did not progress after the 4 cycles of chemotherapy plus immunotherapy, and who did not experience severe immune-related toxicity. Maintenance immunotherapy is administered until disease progression or toxicity [8, 9].

The specific selection of second-line therapy is currently defined by the timeframe between first-line treatment initiation and relapse. Patients who relapse within ≥ 3 months [1] or within ≥ 6 months [2] after first-line therapy are considered chemotherapy-sensitive, and are hence recommended to retreat with the original regimen. However, the use of immune checkpoint inhibitors (ICIs) is discouraged in these patients who relapse while on maintenance with atezolizumab or durvalumab [2]. Of note, a minority of patients experience a long-term benefit from immunotherapy, and there are currently no biomarkers that predict prolonged response to immunotherapy in SCLC [12, 13].

Although the emergence of ICIs has considerably changed the treatment landscape of ES-SCLC in recent years, there is still a gap in evidence on how the use of ICIs is integrated into clinical practice for the therapeutic management of patients with ES-SCLC. Accordingly, and in light of the most recent guidelines on ES-SCLC [1, 2], we designed this large study based on both a physician practice survey and real-world patient data obtained through a chart review approach. The purpose of the present study was twofold: (1) to evaluate diagnosis and treatment practices in ES-SCLC among medical centers located across France specialized in SCLC; and (2) to describe first-line treatment patterns among patients with ES-SCLC following the introduction of immunotherapy into clinical practice. Through this multi-method study, we particularly aimed to better understand patient- and treatment-related factors that might influence the administration of frontline immunotherapy.

## Methods

### Survey-based study component

A collaborative nationwide survey was developed by a scientific committee consisting of 6 oncologists with extensive experience in SCLC management. The 50-item questionnaire was completed by 45 physicians from 45 different medical centers located across France who are involved in the multidisciplinary tumor board in their centers. Responses were collected from June 2022 to January 2023, using SurveyMonkey and through one-hour structured interviews with the surveyed physicians. The physicians were instructed to select answers closest to their own clinical practice. The 45 medical centers were selected in such a way as to ensure adequate geographical distribution and adequate distribution between different types of healthcare facilities. Indeed, the 45 selected medical centers were located in 12 of the 13 regions of Metropolitan France (Corsica being the exception), with the most represented regions being Southwestern France (*n* = 11; 24%), Southeastern France (*n* = 10; 22%), and Paris Region (*n* = 9; 20%). Moreover, 19 medical centers (42%) were general public hospitals, 13 (29%) academic medical centers, 8 (18%) private hospitals, and 5 (11%) non-profit comprehensive cancer centers.

The survey, which was administered in French, mainly consisted of close-ended, multiple-choice questions. An option “other” was also included for several questions, in case the surveyed physicians needed to expand on their answers. Overall, the survey consisted of five sections. In the first section, physicians were asked details about clinical experience in their medical centers, including the number of patients with ES-SCLC cared for annually. The second section was related to the diagnostic workup of ES-SCLC, including the diagnostic methods used by the medical centers for treatment planning of ES-SCLC, the impact of PD-L1 status on clinical decision-making, the mean time between ES-SCLC diagnosis and treatment initiation, and the search for paraneoplastic syndromes that may arise with SCLC. The third section addressed multidisciplinary care coordination in ES-SCLC, including its impact on treatment initiation, and the exclusion criteria for immunotherapy in combination with chemotherapy. In the fourth section, which was the longest, physicians were asked about their centers’ treatment practices in ES-SCLC, primarily related to first-line chemoimmunotherapy with or without PCI/thoracic radiotherapy and the criteria for the selection of the first-line immunotherapy and chemotherapy regimen, maintenance immunotherapy, and second-line therapy. The fifth and final section addressed the organization of health care in ES-SCLC, and the impact of immunotherapy on care pathways. Supplementary Material 1 provides the full study survey translated into English.

### Chart review study component

In parallel to the physician practice survey, we analyzed aggregated information from 548 adult patients with confirmed ES-SCLC receiving treatment in the same medical centers (44/45 centers), using a chart review approach. The study population included all patients aged 18 or over with a cytological and/or histological diagnosis of ES-SCLC who initiated at one of the participating centers first-line cancer therapy consisting of immunotherapy, chemotherapy, and/or radiotherapy. We excluded patients with initially localized SCLC who later developed metastases. The medical specialists, who were responsible for treatment decisions for patients with ES-SCLC at each study center, reviewed the medical charts of 10 to 15 consecutive eligible patients treated between May 2020 and December 2021. Patients could have been alive or deceased at the time of medical chart review.

Data from the patients’ medical charts were registered in a case report form specifically designed for the study. The study measures included patient age, PS, presence of paraneoplastic syndromes, presence of severe comorbidities, presence of metastases, treatment with corticosteroids at the time of ES-SCLC diagnosis, administered first-line treatment, receipt of maintenance immunotherapy, and receipt of radiotherapy. Given the use of a retrospective chart review approach, survival outcomes such as progression-free survival (PFS) or OS were not evaluated.

### Analysis and ethics

Results from both the survey and the chart review study are presented using descriptive statistics, namely counts and percentages. Missing data were not imputed.

This study was conducted in compliance with the French laws and regulations as well as the Declaration of Helsinki and Good Clinical Practice guidelines. Since our study did not involve the direct participation of human subjects nor individual data, and extracted data were aggregated and anonymized, the need for ethics approval and consent to participate was deemed unnecessary according to national regulations (the French Data Protection Agency [*Commission Nationale de l’Informatique et des Libertés*, CNIL]; https://www.cnil.fr/fr/lanonymisation-des-donnees-un-traitement-cle-pour-lopen-data).

## Results

### Survey-based study component

For over three-quarters of the 45 participating medical centers (*n* = 35; 78%), the estimated number of patients with ES-SCLC treated per year was 11 to 40. A vast majority of study centers declared that patients with ES-SCLC were mostly referred by their general practitioners (*n* = 39; 87%). Regarding diagnostic methods generally used for treatment planning of ES-SCLC, all centers (100%) claimed that they perform chest computed tomography (CT) scan for all their patients (with few exceptions), and 42 (93%) declared that they perform bronchial fibroscopy with bronchial biopsy for a vast majority of their patients. In terms of detection of brain metastases, the most common imaging tests used in the study centers for the majority of patients with ES-SCLC were brain CT scan (*n* = 38; 84%) and brain magnetic resonance imaging (MRI) (*n* = 25; 56%). PD-L1 as a biomarker was not tested by most participating centers (*n* = 36; 80%). By contrast, 42 centers (93%) usually search for paraneoplastic syndromes that may arise with SCLC, in most of their patients.

**Fig. 1:**
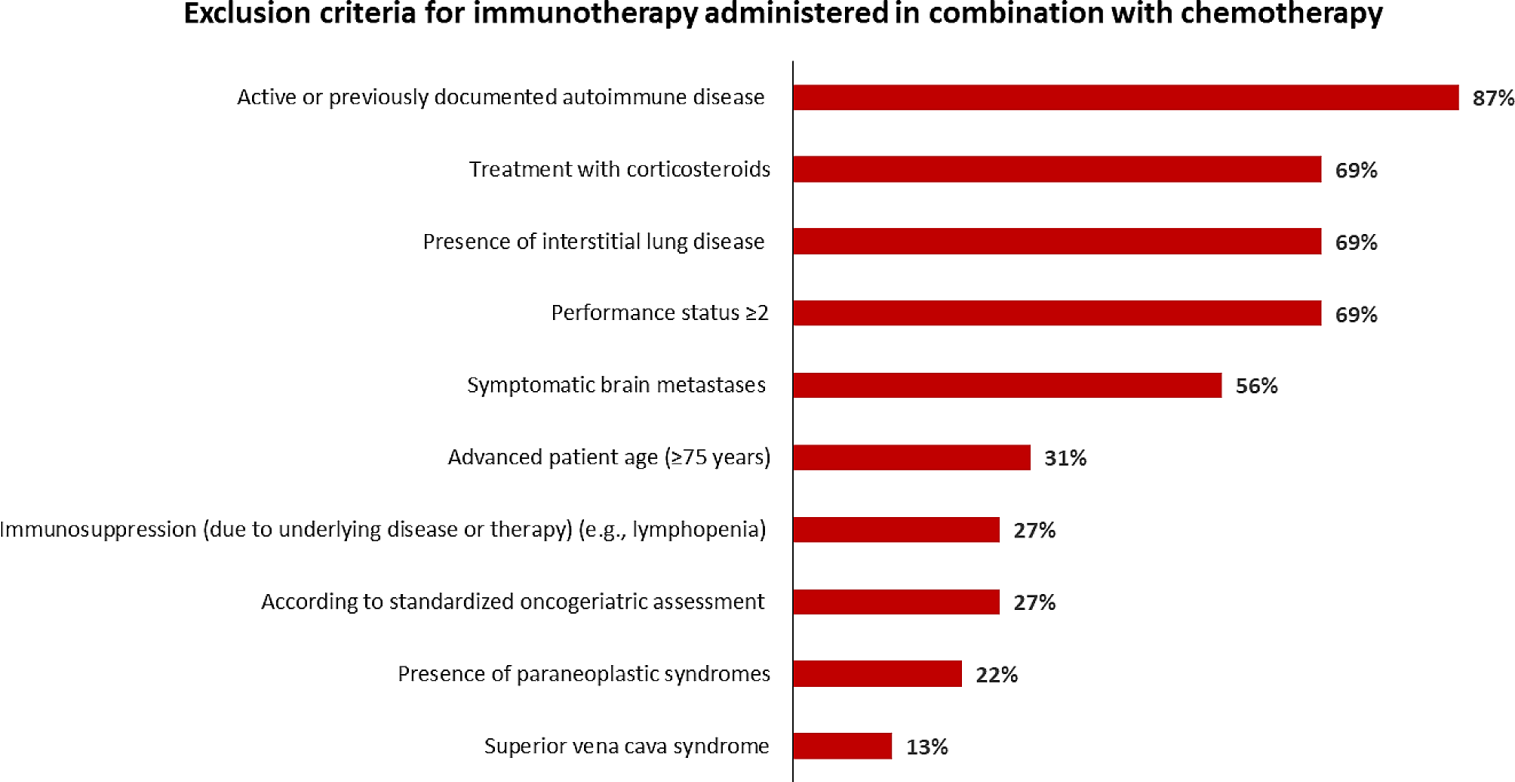
Exclusion criteria for frontline immunotherapy (atezolizumab or durvalumab) in the participating medical centers

The mean time between ES-SCLC diagnosis and treatment initiation ranged between 2 and 7 days, as declared by 37 of the 45 participating medical centers (82%). For almost all centers (*n* = 43; 96%), most of their patients with ES-SCLC were eligible to receive frontline chemoimmunotherapy, and 42/45 centers (93%) would treat the majority of their patients with carboplatin plus etoposide *versus* 3/45 (7%) with cisplatin plus etoposide. The main exclusion criteria for immunotherapy (atezolizumab or durvalumab) in the participating medical centers (Fig. 1) were the presence of autoimmune disease (*n* = 39; 87%), treatment with corticosteroids (*n* = 31; 69%), presence of interstitial lung disease (*n* = 31; 69%), and Eastern Cooperative Oncology Group (ECOG) PS ≥ 2 (*n* = 31; 69%). Regarding the corticosteroid dose threshold (equivalent to mg/day of prednisone), one-third of centers (*n* = 15) considered that a dose of ≥ 20 mg/day is an immunotherapy exclusion criterion. In almost all centers (*n* = 44; 98%), immunotherapy was introduced during cycle 1 of chemotherapy for the majority of patients with ES-SCLC. For patients deemed ineligible to receive immunotherapy, the centers indicated that immunotherapy could be initiated during cycle 2 of chemotherapy if corticosteroids were deintensified/discontinued (*n* = 33; 73%) or in case of PS improvement (*n* = 22; 49%).

**Fig. 2:**
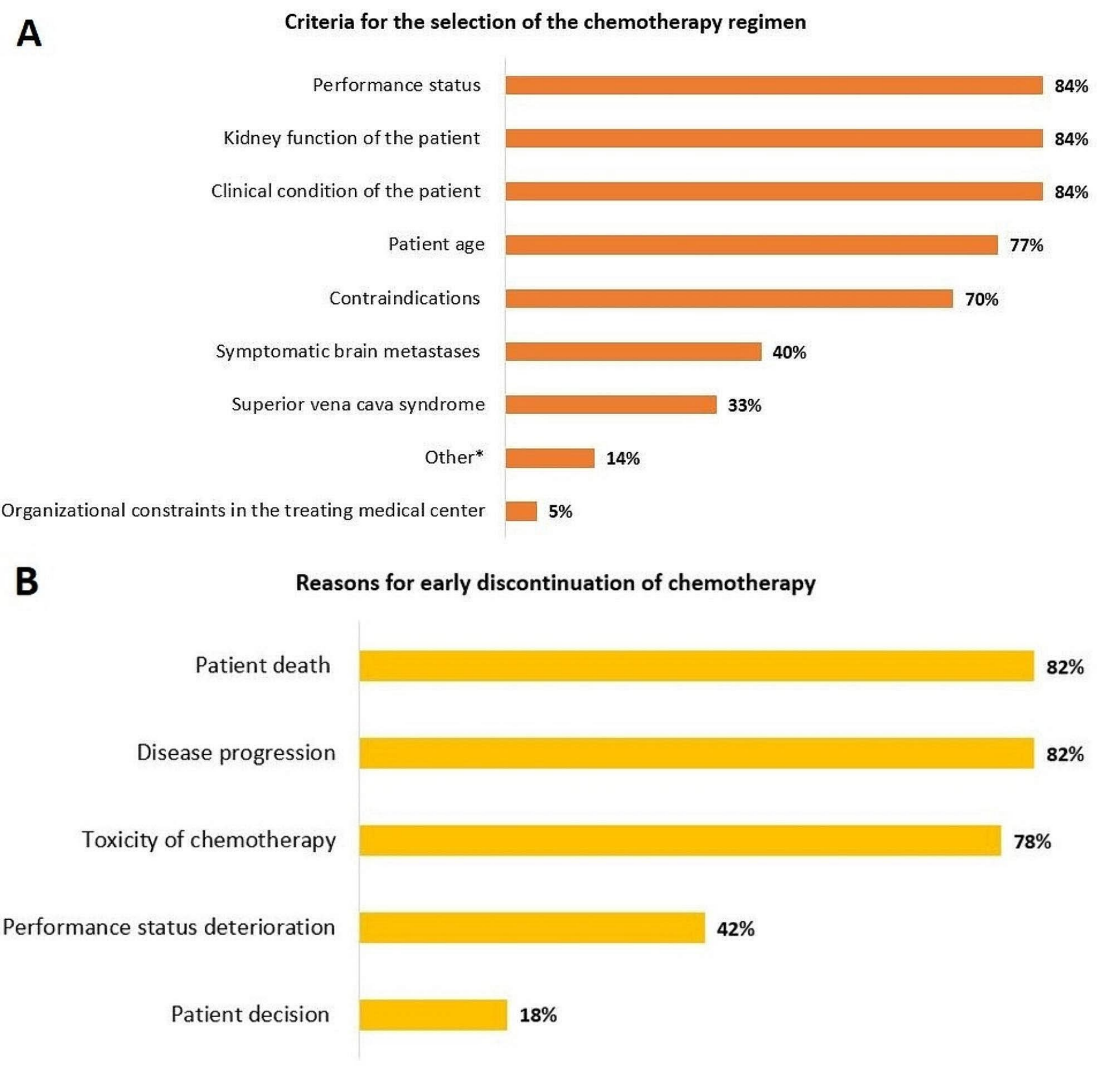
Criteria for the selection of the frontline chemotherapy regimen (carboplatin plus etoposide *versus* cisplatin plus etoposide) to be administered in combination with immunotherapy (**A**), and reasons for early discontinuation of chemotherapy in the participating medical centers (**B**). *Other include: prescribing practices; a decision to administer carboplatin over cisplatin because its use is approved in combination with both atezolizumab and durvalumab; treatment tolerance

Figure 2A illustrates the criteria for the selection of the chemotherapy regimen to be combined with frontline immunotherapy. In the 45 medical centers, the selection of the frontline chemotherapy regimen (carboplatin plus etoposide *versus* cisplatin plus etoposide) was primarily guided by the patient’s PS, kidney function, and clinical condition (all *n* = 38; 84%). The main reasons for early discontinuation of chemotherapy (i.e., before completion of 4 cycles of chemotherapy) (Fig. 2B) were patient death (*n* = 37; 82%), disease progression (*n* = 37; 82%), and toxicity of chemotherapy (*n* = 35; 78%).

**Fig. 3:**
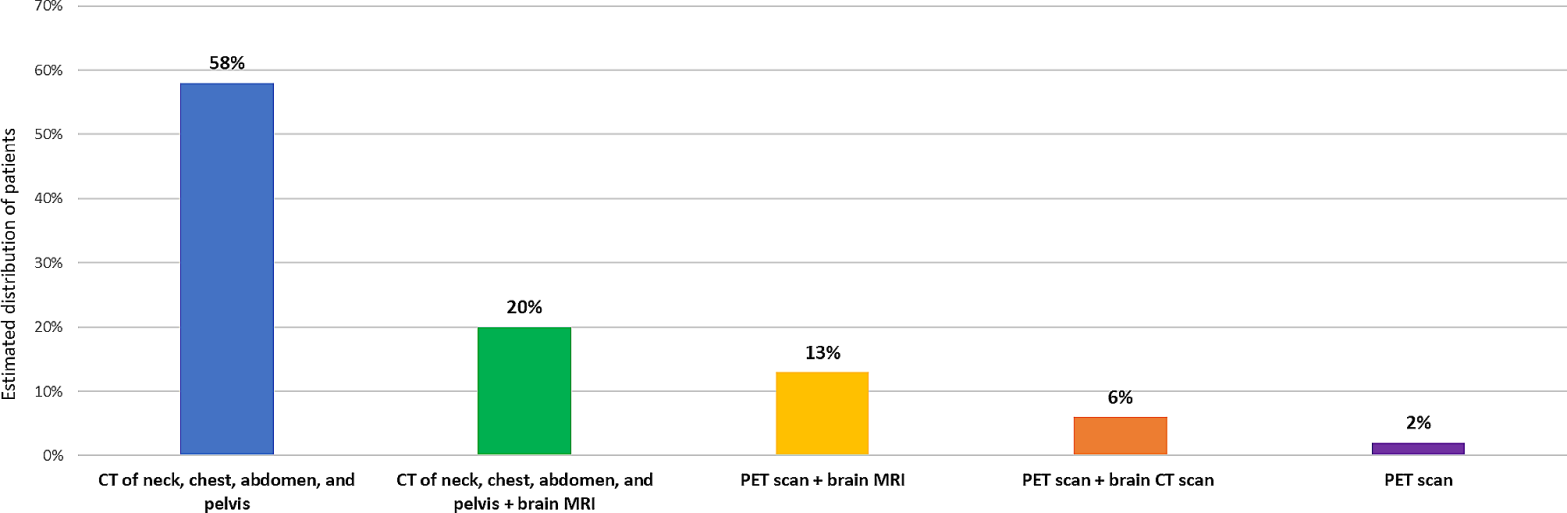
Imaging tests usually performed in the participating medical centers to assess the response to systemic therapy and to monitor metastases in patients with extensive-stage small cell lung cancer. Abbreviations: CT, computed tomography; MRI, magnetic resonance imaging; PET, positron emission tomography

Disease evaluation was most commonly conducted every 8 weeks (as indicated by 12 centers; 27%), 9 weeks (*n* = 16; 36%), or 12 weeks (*n* = 11; 24%). CT of the neck, chest, abdomen, and pelvis was the most frequently performed imaging test to assess the response to systemic therapy and to monitor metastases, whereas the surveyed medical centers performed brain CT in 6% of their patients (Fig. 3). Among the 45 participating medical centers, 24 (53%) and 16 (36%) considered that patients are chemotherapy-sensitive if they relapse within ≥ 3 months and within ≥ 6 months after first-line therapy, respectively. In the second-line setting, most centers (41/45; 91%) indicated that they would retreat the majority of chemotherapy-sensitive patients with the original chemotherapy regimen. However, 36 centers (80%) mentioned that they do not reintroduce or continue immunotherapy among chemotherapy-sensitive patients with relapsed ES-SCLC.

**Fig. 4:**
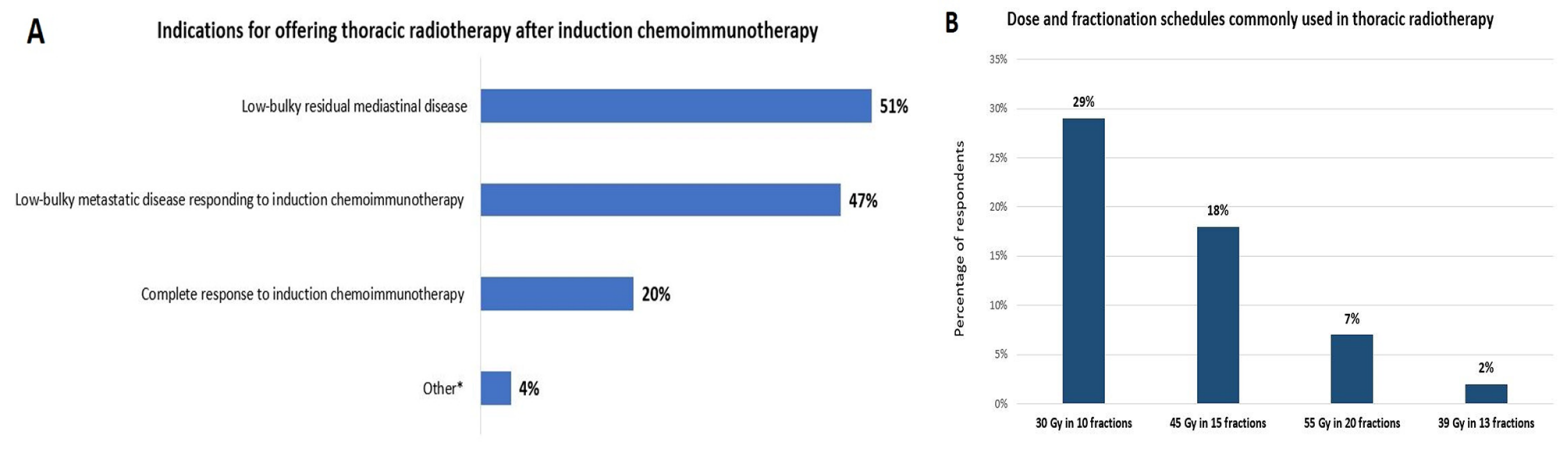
Indications and influencing factors in the participating medical centers for offering thoracic radiotherapy following induction chemoimmunotherapy (**A**), and dose and fractionation schedules commonly used in thoracic radiotherapy for extensive-stage small cell lung cancer (ES-SCLC) (**B**). *Other include: aggressive mediastinal mass at ES-SCLC diagnosis; patients with a metastasis in complete remission but showing persistent disease on positron emission tomography

Three centers (7%) declared that they offer PCI to the majority of their patients, and no centers offer thoracic radiotherapy to the majority of their patients. More than half of the centers (*n* = 23; 51%) would however offer thoracic radiotherapy following induction chemoimmunotherapy if low-bulky residual mediastinal disease was detected (Fig. 4A). The radiotherapy dose and fractionation schedule most commonly used in the medical centers was 30 Gy in 10 once-daily fractions (*n* = 13; 29%) (Fig. 4B).

**Fig. 5:**
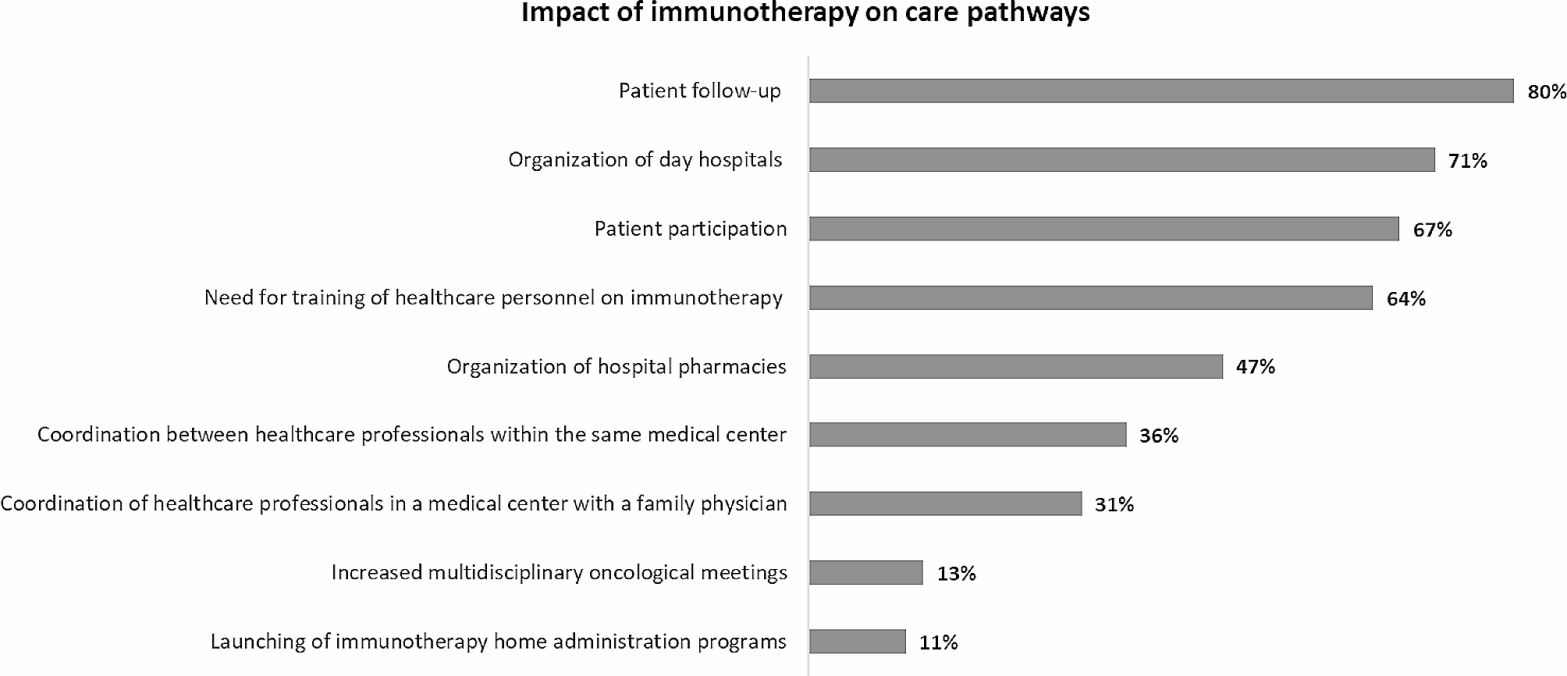
Impact of immunotherapy on care pathways in the participating medical centers

In terms of the impact of immunotherapy on care pathways, over half of the 45 participating medical centers expressed that immunotherapy especially affected patient participation, patient follow-up, organization of day hospitals, and healthcare personnel training (Fig. 5).

### Chart review study component

Aggregated information from a total of 548 patients with ES-SCLC who initiated first-line treatment was included in the chart review study component. Table 1 summarizes patient demographics and clinical characteristics of the study population. Overall, most patients (383/546; 70%) had an ECOG PS ≤ 1, with only 51/545 patients (9%) presenting paraneoplastic syndromes. The vast majority of patients (509/537; 95%) had metastases. These metastases were symptomatic in 272 patients (272/509; 53%), and only 33 of the 509 patients with metastases (6%) reported isolated brain metastasis. Overall, 117 of 524 evaluated patients (22%) were receiving corticosteroids at the time of ES-SCLC diagnosis, among whom only 26 (22%) discontinued their corticosteroid treatment prior to the start of first-line therapy of ES-SCLC.

**Table 1:** Clinical characteristics of the included patients at the time of diagnosis of extensive-stage small cell lung cancer (ES-SCLC)

Variable		n/N (%)
Age, years	< 65	231/548 (42.2)
	65–75	211/548 (38.5)
	> 75	106/548 (19.3)
Eastern Cooperative Oncology Group (ECOG) performance status	0	103/546 (18.9)
	1	280/546 (51.3)
	2	112/546 (20.5)
	> 2	51/546 (9.3)
Presence of paraneoplastic syndromes		51/545 (9.4)
Presence of at least one severe comorbidity†		149/545 (27.3)
Presence of metastasis	None	28/537 (5.2)
	Symptomatic brain metastasis only	20/537 (3.7)
	Asymptomatic brain metastasis only	13/537 (2.4)
	Multi-organ metastases including symptomatic brain metastasis	252/537 (46.9)
	Multi-organ metastases including asymptomatic brain metastasis	224/537 (41.7)
Corticosteroid use before initiation of first-line therapy		117/524 (22.3)

†Included severe comorbidities were: chronic obstructive pulmonary disease (COPD) classified according to the Global Initiative for Obstructive Lung Disease (GOLD) as GOLD 3 (severe) or GOLD 4 (very severe); interstitial lung disease with a transfer factor for carbon monoxide (TLCO) > 70% or 50–70%; congestive heart failure; complicated diabetes; moderate to severe renal impairment; moderate to severe hepatic impairment; hemopathy; use of immunosuppressant medications

n refers to the number of evaluated patients for each parameter, and N refers to the total number of patients with available information

First-line treatment patterns in the study population are presented in Table 2. Among 548 evaluated patients, 409 (75%) received chemoimmunotherapy as a first-line treatment of ES-SCLC, 374 (91%) of whom carboplatin plus etoposide and 35 (9%) cisplatin plus etoposide. Of the 409 patients who received chemoimmunotherapy as a first-line treatment, 379 (93%) had their immunotherapy introduced during cycle 1 of chemotherapy. In total, 89% of patients who initiated chemoimmunotherapy as a first-line treatment received at least 4 cycles of platinum-etoposide chemotherapy *versus* 69% among those who initiated chemotherapy alone as first-line treatment. Overall, 340/548 patients (62%) received maintenance immunotherapy. Of them, 14% received maintenance immunotherapy for at least 9 months, 58% for at least 3 months, and 42% for less than 3 months. Regarding radiotherapy use in the first-line setting, most patients (367/539; 68%) did not receive radiotherapy of any type or PCI.

**Table 2:** First-line treatment patterns for the included patients with extensive-stage small cell lung cancer (ES-SCLC)

Treatment received		n/N (%)
Chemotherapy alone	Carboplatin + etoposide	114/548 (20.8)
	Cisplatin + etoposide	9/548 (1.6)
Chemoimmunotherapy	Atezolizumab + carboplatin + etoposide	252/548 (46.0)
	Durvalumab + carboplatin + etoposide	122/548 (22.3)
	Durvalumab + cisplatin + etoposide	35/548 (6.4)
Other		16/548 (2.9)
Number of administered chemotherapy cycles	1	35/530 (6.6)
	2	25/530 (4.7)
	3	25/530 (4.7)
	4	284/530 (53.6)
	5	21/530 (4.0)
	6	124/530 (23.4)
	> 7	16/530 (3.0)
Receipt of maintenance immunotherapy		340/548 (62.0)
Receipt of radiotherapy	None	365/539 (67.9)
	Brain radiotherapy alone	103/539 (19.1)
	Thoracic radiotherapy	34/539 (6.3)
	Brain radiotherapy + thoracic radiotherapy	18/539 (3.3)
	Prophylactic cranial irradiation	18/539 (3.3)

n refers to the number of evaluated patients for each parameter, and N refers to the total number of patients with available information

## Discussion

In this multi-method, nationwide, real-world study from France, we analyzed the clinical management patterns of ES-SCLC using aggregated information from 548 patients with ES-SCLC who initiated first-line therapy, coupled with a physician practice survey performed among 45 medical centers. Our findings show an overall alignment of practices among the French medical centers managing patients with ES-SCLC as well as consistency with established, evidence-based guidelines [1, 2]. Indeed, all participating centers surveyed in the present study embraced these recent recommendations, establishing chemoimmunotherapy as a first-line treatment for most of their patients. Overall, 75% of the 548 patients with ES-SCLC received chemoimmunotherapy as a first-line treatment.

The other prominent observation from the present study is the notable number of patients who reported long-term maintenance immunotherapy. In both IMpower133 [8] and CASPIAN [9], trials that established chemoimmunotherapy as the preferred initial treatment for ES-SCLC, patients continued maintenance therapy with ICIs until disease progression or other discontinuation criteria were met. Consistently, in our chart review study, over half of the study population (58%) received maintenance immunotherapy for at least 3 months. Due to the aggressive nature of ES-SCLC, such results are encouraging for a better understanding of the course and duration of ICIs in treating ES-SCLC. Indeed, it has been suggested that maintenance therapy with an ICI may help increase the durability of therapeutic responses, as sequential use of immunotherapy after chemotherapy can minimize T-cell killing by chemotherapy and extend the effect of ICIs to the furthest [14].

To realize the full potential of ICIs in ES-SCLC, understanding their efficacy and safety in diverse patient populations is critical, including in patients with an ECOG PS ≥ 2 [15]. In the chart review analysis of our study, 30% of the study population had a ECOG PS ≥ 2. By contrast, in the pivotal IMpower133 [8] and CASPIAN [9] trials, a PS ≥ 2 was mentioned as a key exclusion criterion. Other exclusion criteria included: a history of radiotherapy to the chest or planned consolidation chest radiotherapy; active or previous autoimmune or inflammatory disorders; paraneoplastic syndromes of autoimmune nature requiring systemic treatment; previous treatment with ICIs; uncontrolled intercurrent illness such as active infection or interstitial lung disease; current or recent use of immunosuppressive medication such as corticosteroids. Of note, patients with brain metastases and advanced age were not excluded from IMpower133 [8] and CASPIAN [9], for which subgroup analyses showed an OS benefit of chemoimmunotherapy in patients with and without brain metastases and in patients aged < 65 years and ≥ 65 years [8, 9]. Similarly, in IFCT-1905 CLINATEZO, a nationwide, non-interventional, retrospective chart review study from France of 518 patients with ES-SCLC who received atezolizumab plus chemotherapy, median OS was not different in patients with or without baseline brain metastases (9.9 and 11.6 months, respectively) [16]. However, age was a prognostic factor, with patients aged ≤ 65 years experiencing a median OS of 13.2 months compared to 9.8 months in those > 65 years (*p* = 0.03) [16]. In our study, 69%, 56%, and 31% of surveyed centers stated that they would not administer immunotherapy in case of a PS ≥ 2, symptomatic brain metastases, and advanced patient age, respectively. This finding is consistent with a retrospective cohort study from Israel of 102 patients treated for ES-SCLC with chemotherapy with or without immunotherapy, in which patients who received chemotherapy alone were older, had more liver metastases, and a poorer PS compared to those who received chemoimmunotherapy [17]. Undoubtedly, the prescription of chemoimmunotherapy in patients with metastases and advanced age remains a matter of debate since definitive conclusions cannot be drawn from IMpower133 [8] and CASPIAN [9], given the explanatory nature of the analyses and the relatively small number of patients across subgroups. Similarly, baseline characteristics, such as PS, may not rule out a beneficial effect for chemoimmunotherapy in ES-SCLC.

The presence of an active or a previous autoimmune disease was also declared as an immunotherapy exclusion criterion by 87% of surveyed centers. However, real-world study data have shown that ICIs may be administered safely to individuals with inactive low-risk autoimmune diseases such as rheumatoid arthritis or psoriasis [15]. Moreover, most immune-related adverse events that occur in patients with autoimmune diseases who are receiving ICIs are mild and can often be managed with corticosteroids, without discontinuing cancer immunotherapy [18, 19].

When asked about the preferred chemotherapy regimen to be administered in combination with frontline immunotherapy, 93% of surveyed centers declared that they would treat the majority of their patients with carboplatin plus etoposide *versus* 7% with cisplatin plus etoposide. This finding was well reflected in our chart review analysis; among 409 patients treated with first-line chemoimmunotherapy, 91% received carboplatin plus etoposide and 9% cisplatin plus etoposide. In routine clinical practice, carboplatin is frequently preferred over cisplatin in the ES-SCLC setting [20]. This preference seems to be mainly related to the product safety profile rather than its efficacy; carboplatin poses less risk of nephrotoxicity, neuropathy, and emesis than cisplatin, whereas cisplatin has a lower risk of hematologic toxicity than carboplatin [20, 21]. Indeed, a meta-analysis of individual patient data from four randomized trials did not show differences in efficacy between cisplatin and carboplatin in the first-line treatment of SCLC [21]. It is also important to note that based on the IMpower133 trial [8], atezolizumab can only be prescribed in combination with carboplatin plus etoposide, and not with cisplatin plus etoposide.

In terms of diagnosis, all 45 medical centers surveyed in the present study perform chest CT scan and 93% perform bronchial biopsy as the main diagnostic methods of ES-SCLC. Consistently, guidelines from both the European Society for Medical Oncology (ESMO) [1] and the National Comprehensive Cancer Network (NCCN) [2] recommend to carry out a contrast-enhanced CT of the chest, abdomen, and pelvis in all patients with SCLC. They also recommend that a SCLC diagnosis should be preferably assessed based on histological examination of a biopsy [1, 2]. For the detection of brain metastases, the most common imaging test used in the study centers was brain CT scan (84%), while the use of brain MRI was reported by 56% of centers. Clinical practice guidelines specify that when ES-SCLC is established, brain imaging using MRI or CT with contrast should be obtained in all patients, with brain MRI preferred over CT due to its higher sensitivity [1, 2]. However, in routine clinical practice, short-time access to MRI can be limited, and there are also contraindications to MRI (e.g., presence of metal implants, drug infusion pumps, obesity) [22].

Given the aggressive nature of ES-SCLC and the rapid doubling time of the disease, the time between disease diagnosis and the start of therapy must be reduced to enable a good therapeutic chance in this patient population [23]. In the present study, the reported mean time between ES-SCLC diagnosis and first-line treatment initiation was relatively short (between 2 and 7 days). This finding is reassuring, particularly since the median time from diagnosis to treatment among patients with stages I–IV SCLC was estimated at 7.5 days in a systematic review of 38 observational studies published between 2010 and 2020 and including adult patients diagnosed with lung cancer including SCLC [23]. For follow-up of therapeutic response, the ESMO [1] recommends CT scans every 2–3 months in patients with ES-SCLC potentially qualifying for further treatments. Additionally, the NCCN [2] recommends brain MRI (preferred) or CT with contrast every 3–4 months during year 1 then every 6 months during year 2. The frequency of surveillance in patients with ES-SCLC decreases during subsequent years because of the declining risk of recurrence [1, 2]. Accordingly, in our study, the surveyed medical centers performed CT of the neck, chest, abdomen, and pelvis in 78% of their patients, with therapeutic responses most commonly evaluated at an interval of 8 weeks (27%), 9 weeks (36%), or 12 weeks (24%).

Although an overall alignment in practice was observed among the surveyed French medical centers, there was divergence in some survey responses. Most notably, among the 45 participating medical centers, 53% and 36% considered that patients are chemotherapy-sensitive if they relapse within ≥ 3 months and within ≥ 6 months after first-line therapy, respectively. This divergence is also reflected in different clinical practice guidelines, as the ESMO guidelines use a cutoff of ≥ 3 months for chemotherapy-sensitive SCLC and < 3 months for chemotherapy-resistant SCLC [1]. This cutoff is increased to 6 months by the NCCN [2]. Unified clinical practice guidelines on this cutoff would better guide second-line treatment of ES-SCLC in real-world settings. Research efforts should also continue to produce a definite threshold for classifying a relapsed SCLC as chemotherapy-sensitive.

Since the emergence of immunotherapy, there has been an overall decrease in the use of mediastinal radiotherapy and PCI after chemoimmunotherapy. This was reflected in our chart review study, as most evaluated patients (68%) did not receive thoracic radiotherapy or PCI. Moreover, 7% of surveyed centers offer PCI to the majority of their patients, whereas no centers offer thoracic radiotherapy to the majority of their patients. Indeed, the role of PCI or consolidation thoracic radiotherapy in combination with immunotherapy is still not well-defined in patients with ES-SCLC due to data paucity [1]. Hence, the use of PCI or radiotherapy in ES-SCLC should be based on the opinion of a multidisciplinary panel. However, a recent real-world study from South Korea, including 89 patients with ES-SCLC treated with carboplatin plus etoposide alone or in combination with atezolizumab, revealed that thoracic radiation was associated with an improved survival and an acceptable safety profile in combination with both chemoimmunotherapy and chemotherapy [24]. The ongoing RAPTOR phase II/III trial (ClinicalTrials.gov identifier: NCT04402788) is testing the addition of radiotherapy to the usual maintenance therapy with atezolizumab *versus* atezolizumab alone for ES-SCLC. Other ongoing studies (NCT04947774; NCT05617963) may provide additional evidence on the role of PCI in the prevention of brain metastasis in this population, particularly since PCI can reduce the risk of brain metastasis in ES-SCLC when chemotherapy is effective [25].

The present study merges insights from a physician practice survey and real-world patient data obtained through a chart review approach, delivering a precise overview of the current treatment landscape of ES-SCLC management. Indeed, the physician practice survey captures valuable insights into the decision-making processes among healthcare providers treating ES-SCLC in France. In addition, our analyzed patient population is diverse, including individuals from different geographical areas and representing various characteristics such as ECOG performance status levels and metastatic status. When compared to existing real-world studies assessing chemoimmunotherapy in patients with ES-SCLC [17, 24], our study is much larger in scale. The present study is further strengthened by a relatively few missing data. This study nevertheless has certain limitations. All survey data were subject to response biases such as social desirability bias. Moreover, we cannot infer whether there are differences in ES-SCLC management between general public hospitals, academic medical centers, private hospitals, and non-profit comprehensive cancer centers. The use of a retrospective chart review approach limits the data collection to specific clinical variables. Hence, patient characteristics such as the TNM stage of the disease were not available. In addition, patient outcomes such as PFS or OS could not be reliably evaluated given the retrospective nature of the chart review study.

## Conclusions

This multi-method study provides an accurate overview of the current treatment landscape of ES-SCLC in France, since the emergence of immunotherapy in 2020. Our findings show an overall alignment of practices among medical centers managing ES-SCLC across France, as well as consistency with the most recent evidence-based guidelines. Guidelines are useful tools that assist clinicians treating patients with ES-SCLC, as they provide timely information on the best clinical practices for ES-SCLC management. Reflecting current clinical practice guidelines, we reported a high prescription rate of immunotherapy in the first-line setting. Further large prospective real-world studies, which include survival data, could expand our knowledge on how to optimize the management of ES-SCLC in routine clinical practice.

### Electronic supplementary material

Below is the link to the electronic supplementary material.

Supplementary Material 1: Data Collection Tools

Supplementary Material 2: The Surveyed 45 Medical Centers

## Data Availability

All data generated or analyzed during this study are included in this article.

## Abbreviations

AJCC: American Joint Committee on Cancer
CT: Computed tomography
ECOG: Eastern Cooperative Oncology Group
ES: Extensive-stage
ESMO: European Society for Medical Oncology
ES-SCLC: Extensive-stage small cell lung cancer
ICI: Immune checkpoint inhibitor
MRI: Magnetic resonance imaging
NCCN: National Comprehensive Cancer Network
OS: Overall survival
PCI: Prophylactic cranial irradiation
PD-L1: Programmed death-ligand 1
PFS: Progression-free survival
PS: Performance status
SCLC: Small cell lung cancer
TNM: Tumor, node, metastasis
