# Supplementary material for: Real-life clinical management patterns in extensive-stage small cell lung cancer across France: a multi-method study
Source: BMC Cancer. 2024 Apr 5;24:421. doi: 10.1186/s12885-024-12117-9 (PMC10996204; doi:10.1186/s12885-024-12117-9)
Supplement: Supplementary file 1 — Supplementary Material 1: Data Collection Tools [file 12885_2024_12117_MOESM1_ESM.docx]

**Supplementary Material 1. Data Collection Tools**

***The 50-item questionnaire completed by physicians from the 45 medical centers***

1. *Professional Experience:*
2. **What is the name of your center?**
3. **How many patients with limited-stage SCLC do you care for annually in your center?**
   - 0-5 patients
   - 6-10 patients
   - 11-15 patients
   - 16-20 patients
   - 21-25 patients
   - More than 25 patients
   - I do not know
4. **How many patients with extensive-stage SCLC do you care for annually in your center?**
   - 0-10 patients
   - 11-20 patients
   - 21-30 patients
   - 31-40 patients
   - 41-50 patients
   - More than 50 patients
   - I do not know
5. **Among your patients with metastatic SCLC, how many patients relapse after being initially diagnosed with limited-stage SCLC?**
   - 0-5 patients
   - 6-10 patients
   - 11-15 patients
   - 16-20 patients
   - 21-25 patients
   - More than 25 patients
   - I do not know
6. **Which healthcare professional(s) refer patients with extensive-stage SCLC to you?**
   - A general practitioner
   - The emergency services
   - A community pulmonologist
   - A pulmonologist from another center
   - A pulmonologist from the pulmonology department of your center
   - An oncologist in another center
   - Other
   - I do not know
7. **To which healthcare professional(s) at your center are patients with suspected extensive-stage SCLC most commonly referred?**
   - A pneumo-oncologist
   - A radiotherapist
   - A surgeon
   - A medical oncologist
   - Other
   - I do not know

1. *Diagnosis:*
2. **If there is a suspected extensive-stage SCLC and considering the urgency of the case, is there a rapid diagnostic protocol within your center?**

|  | Yes, for all patients | Yes, in theory | No | I do not know |
| --- | --- | --- | --- | --- |
| A first consultation with a healthcare provider from the center |  |  |  |  |
| The performance of medical tests |  |  |  |  |

1. **In the diagnostic workup of** **extensive-stage SCLC, what is the proportion of patients for whom you perform the following tests?**

*Several tests have been listed; some may seem irrelevant depending on the surveyed center.*

|  | All with exceptions (96 to 100% of patients) | Vast majority (75 to 95% of patients) | Majority (50 to 74% of patients) | Minority (25 to 49% of patients) | Small minority (6 to 24% of patients) | None with exceptions (0 to 5% of patients) | I do not know |
| --- | --- | --- | --- | --- | --- | --- | --- |
| Chest CT |  |  |  |  |  |  |  |
| Bronchial fibroscopy |  |  |  |  |  |  |  |
| Brain CT |  |  |  |  |  |  |  |
| Brain MRI |  |  |  |  |  |  |  |
| PET scan |  |  |  |  |  |  |  |
| Neuron-specific enolase test |  |  |  |  |  |  |  |
| Myelogram |  |  |  |  |  |  |  |

1. **Does the result of the PD-L1 test impact the** **therapeutic management of your patients with extensive-stage SCLC?**

- Yes
- No
- I do not test for PD-L1 expression
- I do not know

**10. What other biomarkers do you expect to test in the coming years?**

- Molecular subtypes
- Other biomarkers
- None
- I do not know

**11. For a patient with** **a suspected extensive-stage SCLC, what is the mean timeframe between the first consultation, or arrival at the emergency room of your center, and the diagnosis?**

- Less than 4 days
- Between 4 and 7 days
- Between 7 and 14 days
- Between 14 and 21 days
- More than 21 days
- I do not know

**12. What is the mean timeframe between diagnosis and treatment initiation for your patients with extensive-stage SCLC?**

- Less than 2 days
- Between 2 and 5 days
- Between 5 and 7 days
- Between 7 and 14 days
- More than 14 days
- I do not know

**13. Taking into account the urgency of the case,** **what is the proportion of patients with SCLC for whom you would initiate chemotherapy before obtaining the full results of the metastatic workup?**

- 96% to 100% of patients (All with exceptions)
- 75% to 95% of patients (A vast majority)
- 50% to 74% of patients (A majority)
- 25% to 49% of patients (A minority)
- 6% to 24% of patients (A small minority)
- 0% to 5% of patients (None with exceptions)
- I do not know

**14. A delay in which of the following tests impacts the therapeutic chance of your patients with extensive-stage SCLC?**

- Chest CT
- Bronchial fibroscopy
- Brain CT
- Brain MRI
- PET scan
- None
- I do not know

**15.** **As part of the pre-treatment evaluation, what is the proportion of patients with extensive-stage SCLC for whom you search for paraneoplastic syndromes?**

|  | All with exceptions (96 to 100% of patients) | Vast majority (75 to 95% of patients) | Majority (50 to 74% of patients) | Minority (25 to 49% of patients) | Small minority (6 to 24% of patients) | None with exceptions (0 to 5% of patients) | I do not know |
| --- | --- | --- | --- | --- | --- | --- | --- |
| Before the emergence of immunotherapy |  |  |  |  |  |  |  |
| Since the emergence of immunotherapy |  |  |  |  |  |  |  |

1. *Multidisciplinary Care Coordination:*

**16. What is the proportion of patients with extensive-stage SCLC for whom you initiate treatment before their case evaluation at a multidisciplinary tumor board?**

- 96% to 100% of patients (All with exceptions)
- 75% to 95% of patients (A vast majority)
- 50% to 74% of patients (A majority)
- 25% to 49% of patients (A minority)
- 6% to 24% of patients (A small minority)
- 0% to 5% of patients (None with exceptions)
- I do not know

**17. What is the proportion of your patients with extensive-stage SCLC who are eligible to receive immunotherapy in combination with chemotherapy?**

- 96% to 100% of patients (All with exceptions)
- 75% to 95% of patients (A vast majority)
- 50% to 74% of patients (A majority)
- 25% to 49% of patients (A minority)
- 6% to 24% of patients (A small minority)
- 0% to 5% of patients (None with exceptions)
- I do not know

**18.** **For each of the criteria below, indicate whether it can be an exclusion criterion for immunotherapy when combined with chemotherapy for your patients with extensive-stage SCLC:**

|  | Yes | No | Not applicable | I do not know |
| --- | --- | --- | --- | --- |
| Performance status |  |  |  |  |
| Patient age |  |  |  |  |
| Standardized oncogeriatric assessment |  |  |  |  |
| Presence of pre-existing autoimmune disease |  |  |  |  |
| Presence of interstitial lung disease |  |  |  |  |
| Superior vena cava syndrome |  |  |  |  |
| Presence of asymptomatic brain metastases |  |  |  |  |
| Presence of symptomatic brain metastases |  |  |  |  |
| Presence of paraneoplastic syndrome |  |  |  |  |
| Treatment with corticosteroids |  |  |  |  |
| An underlying immunosuppression (e.g., lymphopenia) |  |  |  |  |

**19. For the criteria below, indicate what is the threshold at which your patients with extensive-stage SCLC will no longer be eligible to receive immunotherapy in combination with chemotherapy:**

|  | Threshold | Not applicable | I do not know |
| --- | --- | --- | --- |
| Performance status |  |  |  |
| Patient age |  |  |  |
| Standardized oncogeriatric assessment |  |  |  |
| Maximum dose of corticosteroids (prednisone equivalent in mg/day) |  |  |  |

1. *Treatment Practices:*

*First-Line Therapy*

**20. What proportion of your patients with extensive-stage SCLC, eligible to receive immunotherapy in combination with chemotherapy, did receive this type of treatment?**

- 96% to 100% of patients (All with exceptions)
- 75% to 95% of patients (A vast majority)
- 50% to 74% of patients (A majority)
- 25% to 49% of patients (A minority)
- 6% to 24% of patients (A small minority)
- 0% to 5% of patients (None with exceptions)
- I do not know

**21. Beyond the clinical reasons, what are the reasons** **for which your patients with extensive-stage SCLC, eligible to receive immunotherapy in combination with chemotherapy, do not receive this treatment?**

- Patient refusal
- Patient lost to follow-up
- Barriers to accessing treatment
- Insufficient benefit/risk ratio
- Insufficient ratio of expected benefit/necessary investment
- Patient death
- Other
- I do not know

**22. For your patients with extensive-stage SCLC, what are the criteria for choosing the administered immunotherapeutic agent?**

- Patient’s clinical condition
- Patient’s kidney function
- Patient age
- Patient’s performance status
- Choice of platinum salt
- Treatment efficacy and tolerance
- Guidelines' recommendations
- Accumulated clinical evidence
- Frequency and ease of administration of treatment
- Personal experience/experience of the multidisciplinary tumor board
- Other
- No specific criteria for choosing the immunotherapeutic agent
- I do not know

**23. Among patients with extensive-stage SCLC** **eligible to receive immunotherapy in combination with chemotherapy, what proportion receive immunotherapy?**

|  | All with exceptions (96 to 100% of patients) | Vast majority (75 to 95% of patients) | Majority (50 to 74% of patients) | Minority (25 to 49% of patients) | Small minority (6 to 24% of patients) | None with exceptions (0 to 5% of patients) | I do not know |
| --- | --- | --- | --- | --- | --- | --- | --- |
| Starting the first cycle of chemotherapy |  |  |  |  |  |  |  |
| Starting the second cycle of chemotherapy |  |  |  |  |  |  |  |
| Starting the third cycle of chemotherapy or beyond |  |  |  |  |  |  |  |

**24.** **For patients with extensive-stage SCLC** **ineligible to receive immunotherapy during the first cycle of chemotherapy, what criteria influence the decision to initiate immunotherapy starting the second cycle of chemotherapy?**

- Performance status improvement
- Decrease in tumor size
- Organization of the center
- De-intensified/discontinued corticosteroids
- Other
- Not applicable
- I do not know

**25. For your patients with extensive-stage SCLC, what are the criteria for choosing the administered chemotherapy regimen?**

- Patient’s clinical condition
- Patient’s kidney function
- Superior vena cava syndrome
- Symptomatic brain metastases
- Patient age
- Patient’s performance status
- Contraindications
- Organizational constraints in the center
- Other
- I do not know

**26. Before the emergence of immunotherapy, how were your patients with extensive-stage SCLC** **distributed according to the first-line chemotherapy regimen administered?**

|  | All with exceptions (96 to 100% of patients) | | Vast majority (75 to 95% of patients) | Majority (50 to 74% of patients) | Minority (25 to 49% of patients) | Small minority (6 to 24% of patients) | None with exceptions (0 to 5% of patients) | I do not know |
| --- | --- | --- | --- | --- | --- | --- | --- | --- |
| Carboplatin + etoposide | |  |  |  |  |  |  |  |
| Cisplatin + etoposide | |  |  |  |  |  |  |  |
| Other chemotherapy | |  |  |  |  |  |  |  |

**27. Since the emergence of immunotherapy, how were your patients with extensive-stage SCLC** **distributed according to the first-line chemotherapy regimen administered?**

|  | All with exceptions (96 to 100% of patients) | Vast majority (75 to 95% of patients) | Majority (50 to 74% of patients) | Minority (25 to 49% of patients) | Small minority (6 to 24% of patients) | None with exceptions (0 to 5% of patients) | I do not know |
| --- | --- | --- | --- | --- | --- | --- | --- |
| Carboplatin + etoposide |  |  |  |  |  |  |  |
| Cisplatin + etoposide |  |  |  |  |  |  |  |
| Other chemotherapy |  |  |  |  |  |  |  |

**28. How many cycles of chemotherapy do you perform for the majority of patients with extensive-stage SCLC?**

|  | 2 cycles | 3 cycles | 4 cycles | 5 cycles | 6 cycles | More than 6 cycles | I do not know |
| --- | --- | --- | --- | --- | --- | --- | --- |
| Before the emergence of immunotherapy for patients treated with chemotherapy |  |  |  |  |  |  |  |
| Since the emergence of immunotherapy for patients not receiving immunotherapy (chemotherapy alone) |  |  |  |  |  |  |  |
| Since the emergence of immunotherapy for patients receiving immunotherapy starting the first cycle of chemotherapy |  |  |  |  |  |  |  |
| Since the emergence of immunotherapy for patients receiving immunotherapy starting the second cycle of chemotherapy or beyond |  |  |  |  |  |  |  |

**29.** **What proportion of your patients with extensive-stage SCLC** **do not complete the full chemotherapy cycles initially planned?**

- 96% to 100% of patients (All with exceptions)
- 75% to 95% of patients (A vast majority)
- 50% to 74% of patients (A majority)
- 25% to 49% of patients (A minority)
- 6% to 24% of patients (A small minority)
- 0% to 5% of patients (None with exceptions)
- I do not know

**30. For the majority of your patients with extensive-stage SCLC, what are the reasons for the early discontinuation of chemotherapy?**

- Toxicity of chemotherapy
- Disease progression
- Patient death
- Performance status of the patient
- Patient decision
- Other
- I do not know

**31.** **What is the proportion of patients with extensive-stage SCLC for whom you perform prophylactic cranial irradiation?**

|  | All with exceptions (96 to 100% of patients) | Vast majority (75 to 95% of patients) | Majority (50 to 74% of patients) | Minority (25 to 49% of patients) | Small minority (6 to 24% of patients) | None with exceptions (0 to 5% of patients) | I do not know |
| --- | --- | --- | --- | --- | --- | --- | --- |
| Before emergence of immunotherapy |  |  |  |  |  |  |  |
| Since the emergence of immunotherapy |  |  |  |  |  |  |  |

**32.** **Since the emergence of immunotherapy, at what stage(s) of the treatment plan do you perform prophylactic brain irradiation for patients with extensive-stage SCLC?**

- Between the last course of chemotherapy and the initiation of maintenance immunotherapy
- During the maintenance phase, with continuation of immunotherapy
- During the maintenance phase, with discontinuation of immunotherapy
- Other
- Not applicable
- I do not know

**33. What is the proportion of patients with extensive-stage SCLC for whom you perform consolidative thoracic radiotherapy?**

|  | All with exceptions (96 to 100% of patients) | Vast majority (75 to 95% of patients) | Majority (50 to 74% of patients) | Minority (25 to 49% of patients) | Small minority (6 to 24% of patients) | None with exceptions (0 to 5% of patients) | I do not know |
| --- | --- | --- | --- | --- | --- | --- | --- |
| Before the emergence of immunotherapy |  |  |  |  |  |  |  |
| Since the emergence of immunotherapy |  |  |  |  |  |  |  |

**34. Since the emergence of immunotherapy, at what stage(s) of the treatment plan do you perform the consolidative thoracic radiotherapy for patients with extensive-stage SCLC?**

- Between the last course of chemotherapy and the initiation of maintenance immunotherapy
- During the maintenance phase, with continuation of immunotherapy
- During the maintenance phase, with discontinuation of immunotherapy
- Other
- Not applicable
- I do not know

**35. During a chemotherapy regimen in combination with immunotherapy, what is the proportion of patients with extensive-stage SCLC for whom you perform consolidative thoracic radiotherapy?**

- All patients
- Patients with a low-bulky residual mediastinal disease
- Patients with a complete response
- Patients with a low-bulky metastatic disease responding to systemic chemoimmunotherapy
- Other
- Not applicable
- I do not know

**36. In case of consolidative thoracic radiotherapy, what dose and fractionation schedules do you use for the majority of your patients with extensive-stage SCLC?**

- 30 Gray in 10 fractions
- 39 Gray in 13 fractions
- 45 Gray in 15 fractions
- 55 Gray in 20 fractions
- Other
- I do not know

*Maintenance Immunotherapy*

**37. What is the proportion of your patients with extensive-stage SCLC who benefit from maintenance immunotherapy?**

|  | Less than 3 months | Between 3 and 6 months | Between 6 and 12 months | More than 12 months |
| --- | --- | --- | --- | --- |
| 96% to 100% of patients (all with exceptions) |  |  |  |  |
| 75% to 95% of patients (a vast majority) |  |  |  |  |
| 50% to 74% of patients (a majority) |  |  |  |  |
| 25% to 49% of patients (a minority) |  |  |  |  |
| 6% to 24% of patients (a small minority) |  |  |  |  |
| 0% to 5% of patients (none with exceptions) |  |  |  |  |
| I do not know |  |  |  |  |

**38. In your opinion, how are patients with extensive-stage SCLC distributed according to the frequency of maintenance immunotherapy? (v + w + x + y + z = 100%)**

- Every 2 weeks: v%
- Every 3 weeks: w%
- Every 4 weeks: x%
- Other: y%
- I do not know: z%

**39. In your opinion, how are patients with extensive-stage SCLC distributed according to the frequency of overall tumor assessment? (u + v + w + x + y + z = 100%)**

- Every 6 weeks: u%
- Every 8 weeks: v%
- Every 9 weeks: w%
- Every 12 weeks: x%
- Other: y%
- I do not know: z%

**40. In your opinion, how are patients with extensive-stage SCLC distributed according to the test performed to monitor metastases? (u + v + w + x + y + z = 100%)**

- PET scan: u%
- Brain MRI + PET scan: v%
- Brain CT scan + PET scan: w%
- CT of the neck, chest, abdomen, and pelvis: x%
- Brain MRI + CT of the neck, chest, abdomen, and pelvis: y%
- I do not know: z%

**41. Has adverse event monitoring changed since the emergence of immunotherapy?**

- Yes, the frequency of adverse event monitoring is higher
- Yes, the tests performed to monitor adverse effects are different
- No, no change in adverse event monitoring
- Other
- I do not know

*Second-Line Therapy*

**42. What proportion of your patients with extensive-stage SCLC progress?**

|  | Less than 3 months after the last chemotherapy cycle | Between 3 and 6 months after the last chemotherapy cycle | More than 6 months after the last chemotherapy cycle | More than 12 months after the last chemotherapy cycle (long-lasting disease stabilization) |
| --- | --- | --- | --- | --- |
| 96% to 100% of patients (all with exceptions) |  |  |  |  |
| 75% to 95% of patients (a vast majority) |  |  |  |  |
| 50% to 74% of patients (a majority) |  |  |  |  |
| 25% to 49% of patients (a minority) |  |  |  |  |
| 6% to 24% of patients (a small minority) |  |  |  |  |
| 0% to 5% of patients (none with exceptions) |  |  |  |  |
| I do not know |  |  |  |  |

**43. Among your patients with extensive-stage SCLC treated with immunotherapy in combination with chemotherapy,** **what is the mean timeframe between the end of chemotherapy and progression?**

- Less than 1 month
- Between 1 and 2 months
- Between 2 and 3 months
- Between 3 and 6 months
- Between 6 and 12 months
- More than 12 months
- I do not know

**44. What is the timeframe between the end of first-line therapy and progression for you to consider a patient with extensive-stage SCLC as «chemotherapy-sensitive»?**

- 3 months
- 4 months
- 6 months
- Between 6 and 12 months
- More than 12 months
- I do not know

**45. What is the proportion of patients with extensive-stage SCLC, considered as sensitive to first-line chemotherapy, for whom you re-treat with the same chemotherapy regimen in the second-line setting without reintroducing immunotherapy?**

- 96% to 100% of patients (All with exceptions)
- 75% to 95% of patients (A vast majority)
- 50% to 74% of patients (A majority)
- 25% to 49% of patients (A minority)
- 6% to 24% of patients (A small minority)
- 0% to 5% of patients (None with exceptions)
- I do not know

**46. What is the proportion of your patients with extensive-stage SCLC, considered as sensitive to first-line chemotherapy, for whom you reintroduce maintenance immunotherapy in combination with the same chemotherapy regimen in the second-line setting?**

- 96% to 100% of patients (All with exceptions)
- 75% to 95% of patients (A vast majority)
- 50% to 74% of patients (A majority)
- 25% to 49% of patients (A minority)
- 6% to 24% of patients (A small minority)
- 0% to 5% of patients (None with exceptions)
- I do not know

1. *Organization of Health Care:*
2. **What is the proportion of patients with extensive-stage SCLC for whom you propose the following supportive care?**

|  | All with exceptions (96 to 100% of patients) | Vast majority (75 to 95% of patients) | Majority (50 to 74% of patients) | Minority (25 to 49% of patients) | Small minority (6 to 24% of patients) | None with exceptions (0 to 5% of patients) | I do not know |
| --- | --- | --- | --- | --- | --- | --- | --- |
| Adapted physical activity |  |  |  |  |  |  |  |
| Dermatologist *(e.g.,* *rash, pruritus, alopecia, nail damage, …)* |  |  |  |  |  |  |  |
| Dietitian/nutritionist |  |  |  |  |  |  |  |
| Beautician |  |  |  |  |  |  |  |
| Psychologist |  |  |  |  |  |  |  |
| Supportive care medicine: G-CSF |  |  |  |  |  |  |  |
| Supportive care medicine: Antiemetics |  |  |  |  |  |  |  |
| Early palliative care |  |  |  |  |  |  |  |
| Sophrology |  |  |  |  |  |  |  |
| Tobacco cessation services |  |  |  |  |  |  |  |
| Other |  |  |  |  |  |  |  |

1. **In your opinion, what is the proportion of patients with extensive-stage SCLC who do receive the following supportive care?**

|  | All with exceptions (96 to 100% of patients) | Vast majority (75 to 95% of patients) | Majority (50 to 74% of patients) | Minority (25 to 49% of patients) | Small minority (6 to 24% of patients) | None with exceptions (0 to 5% of patients) | I do not know |
| --- | --- | --- | --- | --- | --- | --- | --- |
| Adapted physical activity |  |  |  |  |  |  |  |
| Dermatologist *(e.g.,* *rash, pruritus, alopecia, nail damage, …)* |  |  |  |  |  |  |  |
| Dietitian/nutritionist |  |  |  |  |  |  |  |
| Beautician |  |  |  |  |  |  |  |
| Psychologist |  |  |  |  |  |  |  |
| Supportive care medicine: G-CSF |  |  |  |  |  |  |  |
| Supportive care medicine: Antiemetics |  |  |  |  |  |  |  |
| Early palliative care |  |  |  |  |  |  |  |
| Sophrology |  |  |  |  |  |  |  |
| Tobacco cessation services |  |  |  |  |  |  |  |
| Other |  |  |  |  |  |  |  |

1. **In general, at which time of the patient care trajectory the following supportive care is proposed to your patients with extensive-stage SCLC?**

|  | During announcement of the disease to the patient | During pre-treatment assessment | At treatment initiation | During treatment | At disease progression | At discontinuation of cancer treatments | Other | Not applicable | I do not know |
| --- | --- | --- | --- | --- | --- | --- | --- | --- | --- |
| Adapted physical activity |  |  |  |  |  |  |  |  |  |
| Dermatologist *(e.g.,* *rash, pruritus, alopecia, nail damage, …)* |  |  |  |  |  |  |  |  |  |
| Dietitian/nutritionist |  |  |  |  |  |  |  |  |  |
| Beautician |  |  |  |  |  |  |  |  |  |
| Psychologist |  |  |  |  |  |  |  |  |  |
| Supportive care medicine: G-CSF |  |  |  |  |  |  |  |  |  |
| Supportive care medicine: Antiemetics |  |  |  |  |  |  |  |  |  |
| Early palliative care |  |  |  |  |  |  |  |  |  |
| Sophrology |  |  |  |  |  |  |  |  |  |
| Tobacco cessation services |  |  |  |  |  |  |  |  |  |

1. **What are the parts of the patient care trajectory and the general organization levels of your center on which immunotherapy has an impact?**

- Patient participation
- Need for training of healthcare personnel
- Organization of multidisciplinary tumor boards
- Coordination between healthcare professionals within the center
- Coordination of healthcare professionals within the center with community physicians
- Organization of the day hospital
- Launching of immunotherapy home administration programs
- Organization of the hospital pharmacy
- Patient follow-up
- Other
- None
- I do not know

***The*** ***case report form used for the chart review study component***

1. **How old is the patient?**
   - Less than 65 years
   - Between 65 and 75 years
   - More than 75 years
   - I do not know
2. **What is the patient’s initial performance status?**
   - 0
   - 1
   - 2
   - >2
   - I do not know
3. **Does the patient** **have a paraneoplastic syndrome?**
   - Yes
   - No
   - I do not know
4. **Does the patient** **have at least one of the following severe comorbidities:** **chronic obstructive pulmonary disease (COPD) classified according to the Global Initiative for Obstructive Lung Disease (GOLD) as GOLD 3 (severe) or GOLD 4 (very severe); interstitial lung disease with a transfer factor for carbon monoxide (TLCO) >70% or 50%–70%; congestive heart failure; complicated diabetes; moderate to severe renal impairment; moderate to severe hepatic impairment; hemopathy; use of immunosuppressant medications?**
   - No severe comorbidity from the list presented
   - At least one severe comorbidity from the list presented
   - I do not know
5. **Does the patient** **have metastases?**
   - Symptomatic brain metastasis only
   - Asymptomatic brain metastasis only
   - Symptomatic brain and/or other metastases
   - Asymptomatic brain and/or other metastases
   - None
   - I do not know
6. **Was the patient taking corticosteroids before receiving first-line therapy?**
   - Yes
   - No
   - I do not know
7. **Did the patient discontinue corticosteroids before the initiation of first-line therapy?**
   - Yes
   - No
   - Not applicable
   - I do not know
8. **What first-line treatment did the patient receive?**

- Carboplatin + etoposide
- Cisplatin + etoposide
- Durvalumab + carboplatin + etoposide
- Durvalumab + cisplatin + etoposide
- Atezolizumab + carboplatin + etoposide
- Other
- I do not know

1. **How many cycles of chemotherapy has the patient received?**

- 1 cycle
- 2 cycles
- 3 cycles
- 4 cycles
- 5 cycles
- 6 cycles
- More than 6 cycles
- I do not know

1. **If the patient received immunotherapy, what is the cycle of chemotherapy at which the immunotherapy was introduced?**

- First cycle of chemotherapy
- Second cycle of chemotherapy
- Third cycle of chemotherapy
- Fourth cycle of chemotherapy or beyond
- Not applicable
- I do not know

**11a. Is the patient still on treatment?**

- Yes
- No
- Not applicable
- I do not know

**11b. Did the patient receive maintenance immunotherapy alone?**

***Do not count cycles of immunotherapy in combination with chemotherapy.***

- Yes, <3 months of maintenance immunotherapy
- Yes, between ≥3 and <6 months of maintenance immunotherapy
- Yes, between ≥6 and <9 months of maintenance immunotherapy
- Yes, ≥9 months of maintenance immunotherapy
- No
- Not applicable
- I do not know

1. **Did the patient receive radiotherapy?**
   - Yes, prophylactic cranial irradiation
   - Yes, consolidative thoracic radiotherapy
   - Yes, brain radiotherapy
   - Yes, brain radiotherapy and consolidative thoracic radiotherapy
   - No
   - I do not know
