# Supplementary material for: Real-life clinical management patterns in extensive-stage small cell lung cancer across France: a multi-method study
Source: BMC Cancer. 2024 Apr 5;24:421. doi: 10.1186/s12885-024-12117-9 (PMC10996204; doi:10.1186/s12885-024-12117-9)
Supplement: Supplementary file 2 — Supplementary Material 2: The Surveyed 45 Medical Centers [file 12885_2024_12117_MOESM2_ESM.docx]

**Supplementary Material 2. The Surveyed 45 Medical Centers**

**General public hospitals (n=19)**

| **Center** |
| --- |
| Dax Hospital Center |
| Jura Sud de Lons le Saunier Hospital Center (University Hospital of Besançon) |
| Simone Veil Hospital Center |
| Fleyriat Hospital (Bourg-En-Bresse Hospital) |
| Agen-Nérac Hospital Center |
| Evreux Hospital Center |
| Orléans Regional Hospital Center |
| Chauny Hospital Center |
| Villefranche Hospital Center |
| Tenon Hospital, APHP |
| Saint Musse Hospital, Toulon Hospital Center |
| Ambroise Paré Hospital, APHP |
| Libourne Hospital Center |
| Le Mans Hospital Center |
| European Hospital Georges Pompidou, APHP |
| Chambery Hospital Center |
| Intercommunal Hospital of Créteil |
| Saint Louis Hospital, APHP |
| Broussais Hospital Center (Saint Malo) |

**Academic medical centers (n=13)**

| **Center** |
| --- |
| Brest University Hospital |
| Nimes University Hospital |
| Dijon University Hospital |
| Marseille University Hospital, APHM |
| Rouen University Hospital |
| Toulouse University Hospital, IUCTO |
| Poitiers University Hospital |
| Montpellier University Hospital |
| Strasbourg University Hospital |
| Nantes University Hospital |
| Bicêtre Hospital, APHP |
| Lyon Sud Hospital |
| Limoges University Hospital |

**Private hospitals (n=8)**

| **Center** |
| --- |
| Polyclinic Gentilly |
| Clinique de l’Europe |
| Marzet Clinic |
| Saint Anne Clinic |
| Teissier Clinic, Valenciennes |
| Belharra Clinic |
| Medipole |
| Saint Joseph Hospital |

**Non-profit comprehensive cancer centers (n=5)**

| **Center** |
| --- |
| Léon Bérard Center |
| Cancerology Institute of Lorraine, ICL |
| CLCC Jean Perrin |
| Institut du Thorax (Curie) |
| Cancer Institute of Montpellier, ICM |
